# Systematic Parameter Reviews in Cognitive Modeling: Towards a Robust and Cumulative Characterization of Psychological Processes in the Diffusion Decision Model

**DOI:** 10.3389/fpsyg.2020.608287

**Published:** 2021-01-21

**Authors:** N.-Han Tran, Leendert van Maanen, Andrew Heathcote, Dora Matzke

**Affiliations:** ^1^Department of Human Behavior, Ecology and Culture, Max Planck Institute for Evolutionary Anthropology, Leipzig, Germany; ^2^Department of Experimental Psychology, Utrecht University, Utrecht, Netherlands; ^3^Department of Psychology, University of Tasmania, Hobart, TAS, Australia; ^4^Psychological Methods, Department of Psychology, University of Amsterdam, Amsterdam, Netherlands

**Keywords:** Bayesian inference, cognitive modeling, diffusion decision model, prior distributions, cumulative science

## Abstract

Parametric cognitive models are increasingly popular tools for analyzing data obtained from psychological experiments. One of the main goals of such models is to formalize psychological theories using parameters that represent distinct psychological processes. We argue that systematic quantitative reviews of parameter estimates can make an important contribution to robust and cumulative cognitive modeling. Parameter reviews can benefit model development and model assessment by providing valuable information about the expected parameter space, and can facilitate the more efficient design of experiments. Importantly, parameter reviews provide crucial—if not indispensable—information for the specification of informative prior distributions in Bayesian cognitive modeling. From the Bayesian perspective, prior distributions are an integral part of a model, reflecting cumulative theoretical knowledge about plausible values of the model's parameters (Lee, 2018). In this paper we illustrate how systematic parameter reviews can be implemented to generate informed prior distributions for the Diffusion Decision Model (DDM; Ratcliff and McKoon, 2008), the most widely used model of speeded decision making. We surveyed the published literature on empirical applications of the DDM, extracted the reported parameter estimates, and synthesized this information in the form of prior distributions. Our parameter review establishes a comprehensive reference resource for plausible DDM parameter values in various experimental paradigms that can guide future applications of the model. Based on the challenges we faced during the parameter review, we formulate a set of general and DDM-specific suggestions aiming to increase reproducibility and the information gained from the review process.

## 1. Introduction

With an expanding recent appreciation of the value of quantitative theories that make clear and testable predictions (Lee and Wagenmakers, 2014; Oberauer and Lewandowsky, 2019; Navarro, 2020), cognitive models have become increasingly popular. As a consequence, open science and reproducibility reforms have been expanded to include modeling problems. Lee et al. (2019) proposed a suite of methods for robust modeling practices largely centred on the pre- and postregistration of models. In the interest of cumulative science, we believe that the development and assessment of cognitive models should also include systematic quantitative reviews of the model parameters. Several model classes, including multinomial processing trees (Riefer and Batchelder, 1988), reinforcement learning models (Busemeyer and Stout, 2002), and evidence-accumulation models (Donkin and Brown, 2018), have now been applied widely enough that sufficient information is available in the literature to arrive at a reliable representation of the distribution of the parameter estimates. In this paper, we describe a systematic parameter review focusing on the latter class of models.

A systematic quantitative characterization of model parameters provides knowledge of the likely values of the model parameters and has various benefits. First, it can promote more precise and realistic simulations that help to optimally calibrate and design experiments, avoiding unnecessary experimental costs (Gluth and Jarecki, 2019; Heck and Erdfelder, 2019; Kennedy et al., 2019; Pitt and Myung, 2019; Schad et al., 2020). Second, knowledge about the parameter space can be crucial in maximum-likelihood estimation where an informed guess of the starting point of optimization is often key to finding the globally best solution (Myung, 2003). Third,—and most important for the present paper—systematic quantitative parameter reviews provide crucial information for the specification of informative prior distributions in Bayesian cognitive modeling. The *prior distribution* is a key element of Bayesian inference; it provides a quantitative summary of the likely values of the model parameters in the form of a probability distribution. The prior distribution is combined with the incoming data through the likelihood function to form the *posterior distribution*. The prior distribution is an integral part of Bayesian models, and should reflect theoretical assumptions and cumulative knowledge about the relative plausibility of the different parameter values (Vanpaemel, 2011; Vanpaemel and Lee, 2012; Lee, 2018). Prior distributions play a role both in parameter estimation and model selection. By assigning relatively more weight to plausible regions of the parameter space, informative prior distributions can improve parameter estimation, particularly when the data are not sufficiently informative, for instance due to a small number of observations. Even as the number of observations grows, informative priors remain crucial for Bayesian model selection using Bayes factors (Jeffreys, 1961; Kass and Raftery, 1995). Unfortunately, the theoretical and practical advantages of the prior have been undermined by the common use of vague distributions (Trafimow, 2005; Gill, 2014).

The goal of this paper is to illustrate how a systematic quantitative parameter review can facilitate the specification of informative prior distributions. To this end, we first introduce the Diffusion Decision Model (DDM; Ratcliff, 1978; Ratcliff and McKoon, 2008), a popular cognitive model for two-choice response time tasks (see Ratcliff et al., 2016, for a recent review). Using the DDM as a case study, we will then outline how we used a systematic literature review in combination with principled data synthesis and data quantification using distribution functions to construct informative prior distributions. Lastly, based on the challenges we faced during the parameter review, we formulate a set of general and DDM-specific suggestions about how to report cognitive modeling results, and discuss the limitations of our methods and future directions to improve them.

### 1.1. Case Study: The Diffusion Decision Model

In experimental psychology, inferences about latent cognitive processes from two-choice response time (RT) tasks are traditionally based on separate analyses of mean RT and the proportion of correct responses. However, these measures are inherently related to each other in a speed-accuracy trade-off. That is, individuals can respond faster at the expense of making more errors. Evidence-accumulation models of choice RT and accuracy have provided a solution for this conundrum because they allow for the decomposition of speed-accuracy trade-off effects into latent variables that underlie performance (Ratcliff and Rouder, 1998; Donkin et al., 2009a; van Maanen et al., 2019). These models assume that evidence is first extracted from the stimuli and then accumulated over time until a decision boundary is reached and a response initiated. Among the many evidence-accumulation models, the DDM is the most widely applied, not only in psychology, but also in economics and neuroscience, accounting for experiments ranging from decision making under time-pressure (Voss et al., 2008; Leite et al., 2010; Dutilh et al., 2011), prospective memory (Horn et al., 2011; Ball and Aschenbrenner, 2018) to cognitive control (Gomez et al., 2007; Schmitz and Voss, 2012).

**Figure 1:**
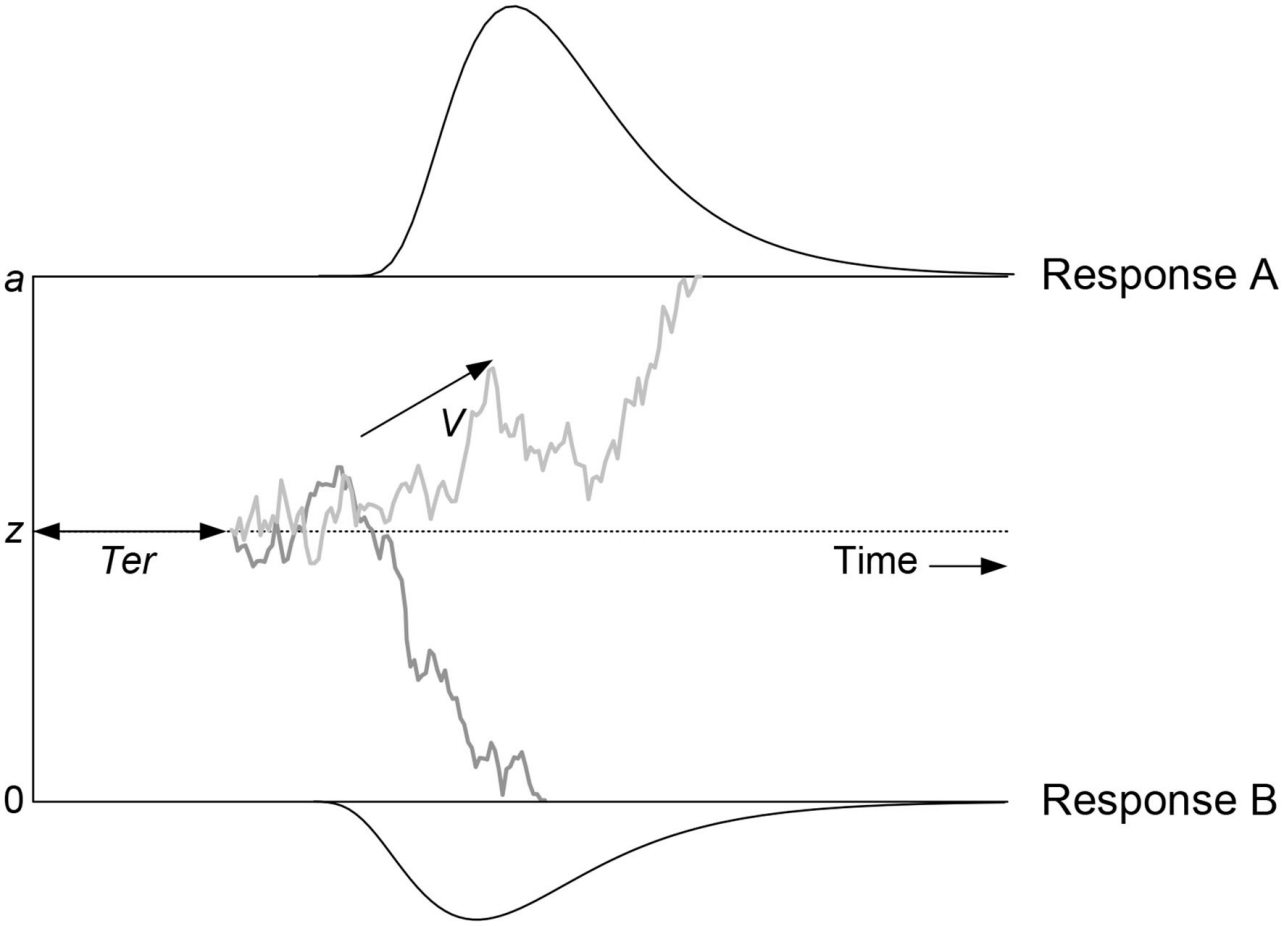
The Diffusion Decision Model (DDM; taken with permission from Matzke and Wagenmakers, 2009). The DDM assumes that noisy information is accumulated over time from a starting point until it crosses one of the two response boundaries and triggers the corresponding response. The gray line depicts the noisy decision process. “Response A” or “Response B” is triggered when the corresponding boundary is crossed. The DDM assumes the following main parameters: drift rate (*v*), boundary separation (*a*), mean starting point (*z*), and mean non-decision time (*T*_*er*_). These main parameters can vary from trial to trial: across-trial variability in drift rate (*s*_*v*_), across-trial variability in starting point (*s*_*z*_), and across-trial variability in non-decision time (*s*_*T*_*er*__). Starting point can be expressed relative to the boundary in order to quantify bias, where $z_{r}=\frac{z}{a}=0.5$ indicates unbiased responding. Similarly, across-trial variability in starting point can be expressed relative to the boundary: *s*_*z*_*r*__ = $\frac{s_{z}}{a}$.

Figure 1 illustrates the DDM. Evidence (i.e., gray line) fluctuates from moment to moment according to a Gaussian distribution with standard deviation *s*, drifting until it reaches one of two boundaries, initiating an associated response. The DDM decomposes decision making in terms of four main parameters corresponding to distinct cognitive processes: (1) the mean rate of evidence accumulation (drift rate *v*), representing subject ability and stimulus difficulty; (2) the separation of the two response boundaries (*a*), representing response caution; (3) the mean starting point of evidence accumulation (*z*), representing response bias; and (4) mean non-decision time (*T*_*er*_), which is the sum of times for stimulus encoding and response execution. RT is the sum of non-decision time and the time to diffuse from the starting point to one of the boundaries. A higher drift rate leads to faster and more accurate responses. However, responses can also be faster because a participant chooses to be less cautious and thus decreases their boundary separation, which will reduce RT but increase errors, causing the speed-accuracy trade-off. Starting accumulation closer to one boundary than the other creates a bias toward the corresponding response. Starting points *z* is therefore most easily interpreted in relation to boundary separation *a*, where the relative starting point, also known as bias, is given by $z_{r}=\frac{z}{a}$. Drift rate can vary from trial to trial according to a Gaussian distribution with standard deviation *s*_*v*_. Both non-decision time and starting point are assumed to be uniformly distributed across trials, with range *s*_*T*_*er*__ and *s*_*z*_, respectively, where *s*_*z*_ can be expressed relative to *a*: *s*_*z*_*r*__ = $\frac{s_{z}}{a}$. One parameter of the accumulation process needs to be fixed to establish a scale that makes the other accumulation-related parameters identifiable (Donkin et al., 2009b). Most commonly this scaling parameters is the moment-to-moment variability of drift rate (*s*), usually with a value fixed to 0.1 or 1.

Fitting the DDM and many other evidence-accumulation models to experimental data is difficult because of the complexity of the models and the form of their likelihood resulting in high correlations among the parameters (i.e., “sloppiness”; Gutenkunst et al., 2007; Gershman, 2016). Informative prior distribution can ameliorate some of these problems. The growing popularity of cognitive modeling has led to extensive application of the DDM to empirical data (Theisen et al., 2020), providing us with a large number of parameter estimates to use for constructing informative prior distributions. In 2009, Matzke and Wagenmakers presented the first quantitative summary of the DDM parameters based on a survey of parameter estimates found in 23 applications. However, their survey is now outdated and was not as extensive or systematic as the approach taken here.

## 2. Materials and Methods

All analyses were written in R or R Markdown (Allaire et al., 2018; R Core Team, 2020). The extraced parameter estimates and the analysis code are available on GitHub (http://github.com/nhtran93/DDM_priors) and the project's Open Science Framework (OSF) site: https://osf.io/9ycu5/.

### 2.1. Literature Search

The literature search was conducted according to the PRISMA guidelines (Moher et al., 2009). Every step was recorded and the inclusion as well as rejection of studies adhered strictly to the pre-specified inclusion criteria. Results from different search engines were exported as BibTex files, maintained with reference management software and exported into separate Microsoft Excel spreadsheets.

#### 2.1.1. Search Queries

The literature search was commenced and completed in December 2017. It consisted of cited reference searches and independent searches according to pre-specified queries. Searches in all databases were preformed three times in order to ensure reproducibility. Four electronic databases were searched with pre-specified queries: (National Library of Medicine, 2017), PsycInfo (American Psychological Association, 2017), Web of Science (WoS, 2017), and Scopus (Elsevier, 2017). A preliminary search of the four databases served to identify relevant search strings, which were different for each database (see the Supplementary Materials or https://osf.io/9ycu5/ for details). The searches began from the publication year of the seminal paper by Ratcliff (1978). The cited reference searches were based on Ratcliff and McKoon (2008), Wiecki et al. (2013), and Palmer et al. (2005), and were performed in both Scopus and Web of Science. These key DDM papers were selected to circumvent assessing an unfeasible number of over 3, 000 cited references to the seminal Ratcliff article, with a potentially high number of false positives (in terms of yielding papers that reported parameter estimates), while still maintaining a wide search covering various areas of psychology and cognitive neuroscience.

#### 2.1.2. Inclusion and Exclusion Criteria

All duplicated references were excluded. After obviously irrelevant papers—judged based on title and abstract—were excluded, the full-texts were acquired to determine the inclusion or exclusion of the remaining articles. Articles were included in the literature review if they (i) used the standard DDM according to Ratcliff (1978) and Ratcliff and McKoon (2008) with or without across-trial variability parameters; and (ii) reported parameter estimates based on empirical data from humans. Articles were excluded if (i) they reported reviews; and (ii) the parameter estimates were based on animal or simulation studies. We also excluded articles that did not report parameter estimates (neither in tables nor in graphs) and articles that estimated parameters in the context of a regression model with continuous predictors that resulted in estimates of intercepts and regression slopes instead of single values of the model parameters.

#### 2.1.3. Data Extraction

The data extraction spreadsheet was pilot-tested using six articles and adjusted accordingly. The following parameter estimates were extracted: drift rate (*v*), boundary separation (*a*), starting point (*z*) or bias ($z_{r}=\frac{z}{a}$), non-decision time (*T*_*er*_), across-trial variability in drift rate (*s*_*v*_), across-trial range in starting point (*s*_*z*_) or relative across-trial starting point (*s*_*z*_*r*__ $=\frac{s_{z}}{a}$), and across-trial range in non-decision time (*s*_*T*_*er*__). Parameter estimates were obtained from tables as well as from graphs using the GraphClick software (Arizona, 2010). Whenever possible, we extracted parameter estimates for each individual participant; otherwise we extracted the mean across participants or in Bayesian hierarchical applications the group-level estimates. When the DDM was fit multiple times with varying parameterizations to the same data within one article, we used the estimates corresponding to the model identified as best by the authors, with a preference for selections made based on the AIC (Akaike, 1973, 1974), in order to identify the best trade-off between goodness-of-fit and parametric complexity (Myung and Pitt, 1997). When the DDM was applied to the same data across different articles, we extracted the parameter estimates from the first application; if the first application did not report parameter estimates, we used the most recent application that reported parameter estimates. Finally, articles that obtained estimates using the EZ (Wagenmakers et al., 2007) or EZ2 (Grasman et al., 2009) methods, or the RWiener R package (Wabersich and Vandekerckhove, 2014), which all fit the simple diffusion model estimating only the four main DDM parameters (Stone, 1960), were excluded due to concerns about potential distortions caused by ignoring across-trial parameter variability (Ratcliff, 2008). Note that we did not automatically exclude all articles without across-trial variability parameters. For articles that did not use EZ, EZ2, or RWiener, but reported models without across-trial variability parameters, we assumed that the author's choice of fixing these parameters to zero was motivated by substantive or statistical reasons and not by the limitations of the estimation software, and hence we included them in the parameter review.

### 2.2. Parameter Transformations

Once extracted, parameter estimates had to be transformed in a way that makes aggregation across articles meaningful. In this section we report issues that arose with respect to these transformations and the solutions that we implemented. A detailed explanation of the transformations can be found in the Supplementary Materials.

#### 2.2.1. Within-Trial Variability of Drift Rate

In all of the studies we examined, the accumulation-related parameters were scaled relative to a fixed value of the moment-to-moment variability in drift rate (typically *s* = 0.1 or *s* = 1). This decision influences the magnitude of all parameter estimates except those related to non-decision time. Once we determined *s* for each article, we re-scaled the affected parameter estimates to *s* = 1. Articles that used the DMAT software (Vandekerckhove and Tuerlinckx, 2008) for parameter estimation were assumed to use the DMAT default of *s* = 0.1, and articles that used HDDM (Wiecki et al., 2013) or fast-DM (Voss and Voss, 2007) were assumed to use the default setting of 1. Articles (co-) authored by Roger Ratcliff were assigned *s* = 0.1.^1^ We excluded 25 articles because the scaling parameter was not reported and even if we assumed the scaling parameter to be *s*, its value could not be determined.

#### 2.2.2. Measurement (RT) Scale

Although the measurement (i.e., RT) scale influences the magnitude of the parameter estimates, none of the articles mentioned explicitly whether the data were fit on the seconds or milliseconds scale. Moreover, researchers did not necessarily report all estimates on the same RT scale. For instance, *T*_*er*_ or *s*_*T*_*er*__ were sometimes reported in milliseconds, whereas the other parameters were reported in seconds. Whenever possible, we used axis labels, captions and descriptions in figures and tables, or the default setting of the estimation software to determine the RT scale. Articles that used the DMAT, HDDM, or fast-DM were assumed to use the default setting of seconds and we assigned an RT scale of seconds to papers authored by Roger Ratcliff ^2^ even if *T*_*er*_ was reported in milliseconds. We also evaluated the plausibility of the reported estimates with respect to the second or millisecond scale by computing a rough estimate of the expected RT for each experimental condition as *E*(*RT*) = (*a* − *z*)/*v*. We then used the following two-step decision rule to determine the RT scale of each parameter:

1. Determine the RT scale of *T*_*er*_: If estimated *T*_*er*_ was smaller than 5, we assumed that *T*_*er*_ was reported in seconds; otherwise we assumed that *T*_*er*_ was reported in milliseconds.
2. Determine RT scale of remaining parameters: If *E*(*RT*) was smaller than 10, we assumed that the remaining parameters were reported in seconds; otherwise we assumed that the remaining parameters were reported in milliseconds.

Once we determined the RT scale for each parameter, we re-scaled the parameter estimates to the seconds scale. Individual parameters estimates that were considered implausible after the transformation (i.e., outside of the parameter bounds, such as a negative *a*) were checked manually. In particular, we checked for (1) inconsistencies in the magnitude across the parameter estimates within articles (e.g., a value of *a* indicative of seconds vs. a value of *T*_*er*_ indicative of milliseconds); (2) reporting or typographic errors; (3) extraction errors; and (4) errors in determining the measurement scale, which typically reflected the use of non-standard experiments or special populations. In a number of cases we also revisited and whenever necessary reconsidered the assigned value of *s*. We removed all parameter estimates from 13 articles that reported implausible estimates reflecting ambiguous or inconsistent RT scale descriptions or clear reporting errors.

#### 2.2.3. Starting Point and Bias

We expressed all starting point *z* and starting point variability *s*_*z*_ estimates relative to *a*. As the attributions of the response options to the two response boundaries is arbitrary, the direction of the bias (i.e., whether *z*_*r*_ is greater or less than 0.5) is arbitrary. As these attributions cannot be made commensurate over articles with different response options, values of *z*_*r*_ cannot be meaningfully aggregated over articles. As a consequence, bias, *z*_*r*_, and its complement, 1 − *z*_*r*_, are exchangeable for the purpose of our summary. We therefore used both values in order to create a single “mirrored” distribution. This distribution is necessarily symmetric with a mean of 0.5, but retains information about variability in bias.^3^

#### 2.2.4. Drift Rate

There are two ways in which drift rates *v* can be reported. In the first, positive drift rates indicate a correct response (e.g., “word” response to a “word” stimulus and “non-word” response to a “non-word” stimulus in a lexical decision task) and negative rates indicate an incorrect response. In the second, positive drift rates correspond to one response option (e.g., “word” response) and negative rates to the other option (e.g., “non-word”). Here, we adopt the former—accuracy coding—method in order to avoid ambiguity regarding the arbitrary attribution of boundaries to response options. We do so by taking the absolute values of the reported drift rates to construct the prior distribution. Readers who wish to adopt the latter—response coding—method, should appropriately mirror our accuracy-coded priors around 0.

### 2.3. Generating Informative Prior Distributions for the DDM Parameters

After post-processing and transforming the parameter estimates, we combined each parameter type across articles and experimental conditions within each study into separate univariate distributions. We then attempted to characterize these empirical parameter distributions with theoretical distributions that provided the best fit to the overall shape of the distributions of parameter estimates.

#### 2.3.1. Parameter Constraints

In many applications of the DDM, researchers impose constraints on the parameter estimates across experimental manipulations, conditions, or groups, either based on theoretical grounds or the results of model-selection procedures. After extracting all parameters from the best fitting models, we identified parameters that were constrained across within- and between-subject manipulations, conditions, or groups within each study. For the purpose of constructing the prior distributions we only considered these fixed parameters once and did not repeatedly include them in the empirical distributions. For instance, a random dot motion task with three difficulty conditions may provide only one estimate for a constrained parameter (i.e., non-decision time), but three parameters for an unconstrained parameter (i.e., drift rate).

#### 2.3.2. Synthesis Across Articles

Most studies reported parameter estimates aggregated across participants, with only eight reporting individual estimates. Before collapsing them with the aggregated estimates, individual estimates were averaged across participants in each study. Parameter estimates were equally weighted when combined across studies as details necessary for weighting them according to their precision were typically not available. We will revisit this decision in the Discussion.

In the results reported in the main body of this article, we aggregated the parameter estimates across all research domains (e.g., neuroscience, psychology, economics), populations (e.g., low/high socioeconomic status, clinical populations), and tasks (e.g., lexical decision, random dot motion tasks). In the Supplementary Materials, we provide examples of prior distributions derived specifically for two of the most common tasks in our database (i.e., lexical decision and random dot motion tasks) and priors restricted to non-clinical populations. Data and code to generate such task and population-specific priors are available in the open repository, so that interested readers can construct priors relevant to their specific research questions.

#### 2.3.3. Distributions

A full characterization of the distribution of model parameters takes into account not only the parameters' average values and variability but also their correlations across participants (e.g., people with lower drift rates may have higher thresholds) and potentially even their correlations across studies or paradigms using multilevel structures. Although multivariate prior distributions would be optimal to represent correlations across participants, they require individual parameter estimates for the estimation of the covariance matrices. As only eight studies reported individual parameter estimates, we were restricted to use univariate distributions.

We attempted to characterize the aggregated results using a range of univariate distribution functions that respected the parameter types' bounds (e.g., non-decision time *T*_*er*_ must be positive) and provided the best fit to the overall shape of the empirical distributions. We first considered truncated normal, lognormal, gamma, Weibull, and truncated Student's *t* distribution functions. However, in some cases the empirical distributions clearly could not be captured by the univariate distributions and were contaminated by outliers due to non-standard tasks, special populations, and possible reporting errors that we not identified during the post-processing steps. We therefore also considered characterizing the empirical distribution using mixture distributions. Mixtures were chosen from the exponential family of distributions that respected the theoretical bounds of the parameter estimates. In particular we used mixtures of two gamma distributions, and truncated normals mixed with either a gamma, lognormal, or another truncated normal distribution. Specifically, we focused on normal mixtures because we assume a finite variance for the parameters and thus the Gaussian distributions represents the most conservative probability distribution to assign to the parameter distributions (for further information see the principles of maximum entropy; Jaynes, 1988).

The univariate and mixture distributions were fit to the empirical distributions using maximum-likelihood estimation (Myung, 2003), with additional constraints on upper and/or lower bounds. For (mirrored) bias *z*_*r*_ and *s*_*z*_*r*__, which are bounded between 0 and 1, we used univariate truncated normal and truncated *t* distributions on [0, 1]. A lower bound of zero was imposed on all other parameters. We then used AIC weights (wAIC; Wagenmakers and Farrell, 2004) to select the theoretical distributions that struck the best balance between goodness-of-fit and simplicity. A table of the AIC and wAIC values for all fitted univariate and mixture distributions and the code to reproduce this table, can be found in the open repository on GitHub or the OSF.

We propose that the wAIC-selected distributions can be used as informative prior distributions for the Bayesian estimation of the DDM parameters. For simplicity, for parameters where a mixture was the best-fitting distribution, we propose as prior the distribution component that best captures the bulk of the parameter estimates as indicated by the highest mixture weight. We will revisit this choice in the Discussion.

## 3. Results

**Figure 2:**
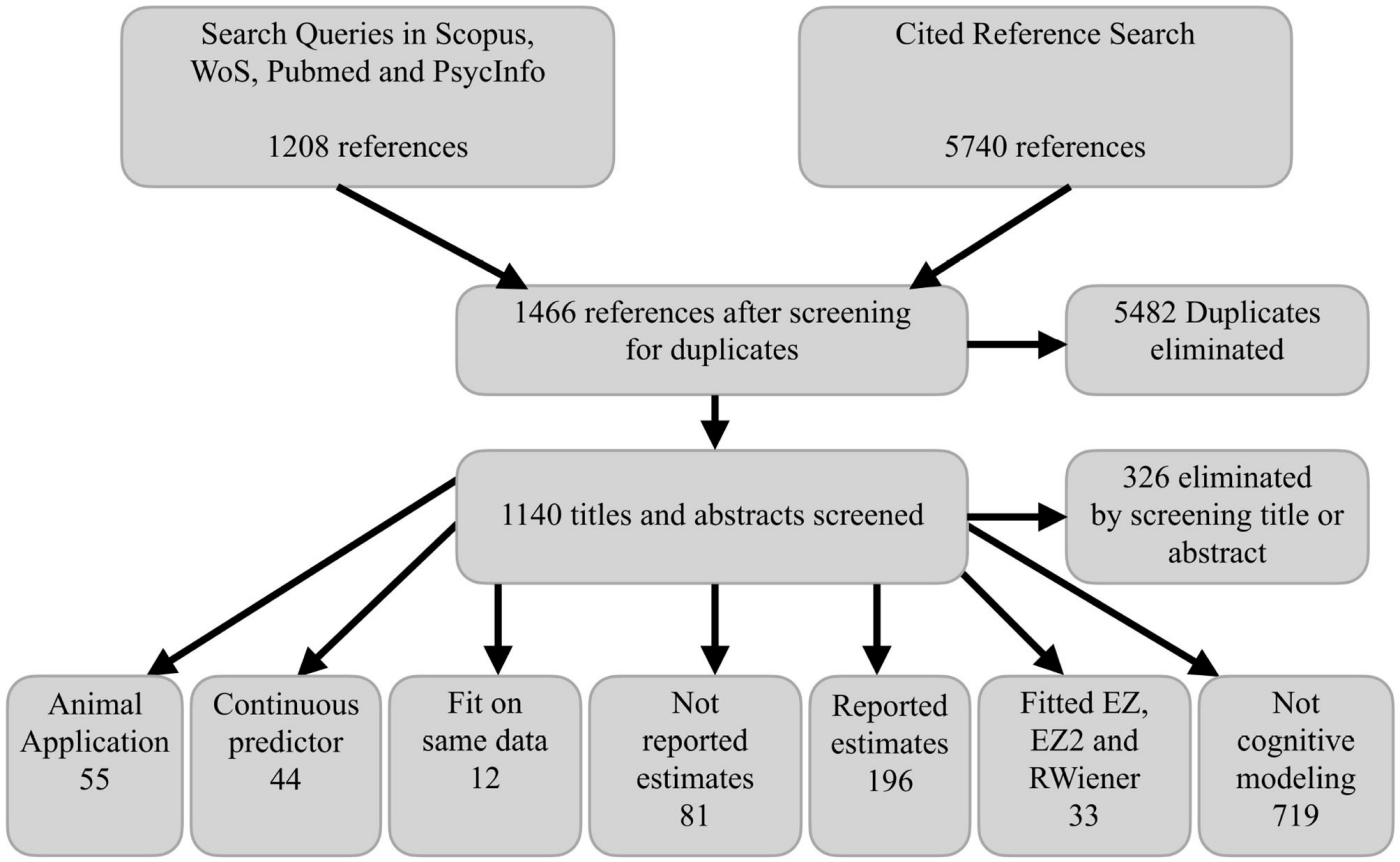
PRISMA flow diagram. WoS, Web of Science. RWiener refers to the R package from Wabersich and Vandekerckhove (2014). EZ and EZ2 refer to estimation methods for the simple DDM developed by Wagenmakers et al. (2007) and Grasman et al. (2009), respectively.

Figure 2 shows the PRISMA flow diagram corresponding to our literature search. The total of 196 relevant articles (i.e., “Reported estimates” in Figure 2) covered a wide range of research areas from psychology and neuroscience to medicine and economics. We excluded 38 references because they did not report the scaling parameters and we were unable to reverse engineer them or because of inconsistent RT scale descriptions or clear reporting errors. Thus, we extracted parameter estimates from a total of 158 references. The most common paradigms were various perceptual decision-making tasks (e.g., random dot motion task; 37 references), lexical decision tasks (33), and recognition memory tasks (17). A total of 29 references included clinical groups and 26 references used Bayesian estimation methods.

**Figure 3:**
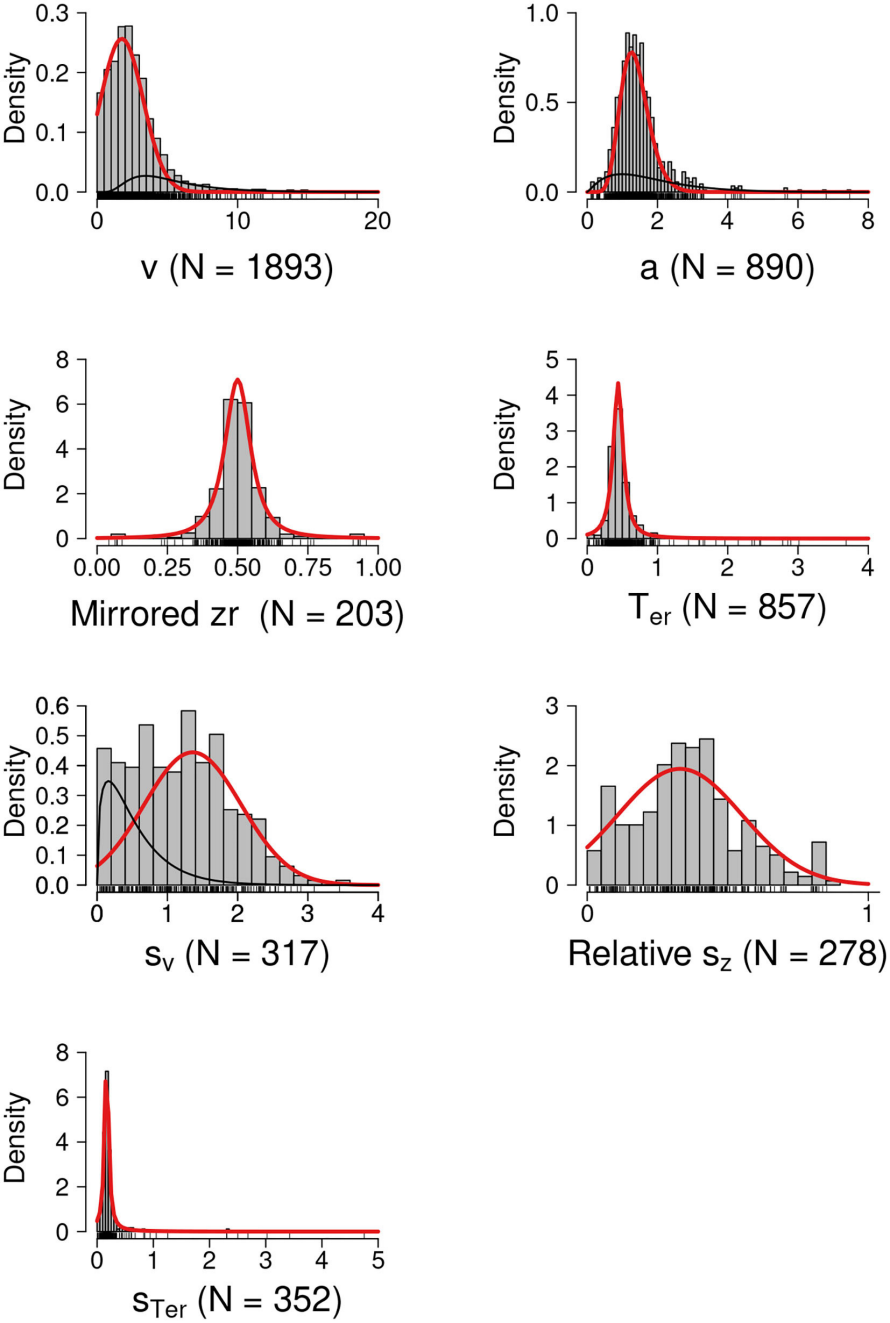
Prior Distributions for the DDM Parameters. The red lines show the best fitting theoretical distributions or the dominant theoretical distribution components with the highest mixture weight (i.e., the proposed informative prior distributions). The black lines show the non-dominant distribution components. *N*, number of unique estimates.

The histograms in Figure 3 show the empirical distributions of the parameter estimates. The red lines show the best fitting theoretical distributions or the dominant theoretical distribution components with the highest mixture weight (i.e., the proposed informative prior distributions). The black lines show the non-dominant mixture distribution components. Note that in most cases the the mixture served to inflate the distributions' tails while preserving a single mode.

Table 1 gives an overview of the informative prior distributions, the corresponding upper and lower bounds (see column “T-LB” and “T-UB”), and whenever appropriate also the mixture weight of the dominant distribution component. The table also shows the upper and lower bounds of the parameter estimates collected from the literature (see column “E-LB” and “E-UB”); these bounds can be used to further constrain parameter estimation by providing limits for prior distributions and bounded optimization methods.

**Table 1:** Informative prior distributions.

**DDM parameter**	***N***	**Distribution**	**Weight**	**Location/Shape**	**Scale**	**df**	**T-LB**	**T-UB**	**E-LB**	**E-UB**
*v*	1,893	Truncated normal^*^ & lognormal	0.85	1.76	1.51		0	+ Inf	0.01	18.51
*a*	890	Gamma^*^ & gamma	0.76	11.69	0.12		0	+ Inf	0.11	7.47
Mirrored *z*_*r*_	203	Truncated t	–	0.5	0.05	1.85	0	1	0.04	0.96
*T*_*er*_	857	Truncated t	–	0.44	0.08	1.32	0	+ Inf	0	3.69
*s*_*v*_	317	Truncated normal^*^ & gamma	0.75	1.36	0.69		0	+ Inf	0	3.45
*s*_*z*_*r*__	278	Truncated normal	–	0.33	0.22		0	1	0.01	0.85
*s*_*T*_*er*__	352	Truncated t	–	0.17	0.04	0.88	0	+ Inf	0	4.75

*N, The number of unique estimates; Weight, The mixture weight of the dominant distribution component; df, Degrees of freedom; T-LB, Theoretical lower bound of the prior distribution; T-UB, Theoretical upper bound of the prior distribution; E-LB, Lower bound of the empirical parameter estimates; E-UB, Upper bound of the empirical parameter estimates*;

*, * Dominant distribution component*.

The results of the model comparisons are available at https://osf.io/9ycu5/. For drift rate *v*, the selected model was a mixture of a zero-bounded truncated normal and a lognormal distribution (wAIC = 0.4), with the mixture weight, and the location and scale of the dominant truncated normal component shown in the first row of Table 1.^4^ For boundary separation *a*, the selected model was a mixture of gamma distributions (wAIC = 0.76), with the shape and scale parameters of the dominant gamma component shown in the second row of Table 1. For non-decision time *T*_*er*_ and across-trial variability in non-decision time *s*_*T*_*er*__, the selected model was a zero-bounded truncated *t* distribution (wAIC = 1 for both *T*_*er*_ and *s*_*T*_*er*__). For mirrored bias *z*_*r*_, the selected model was a truncated *t* distribution on [0, 1] (wAIC = 1.0). For across-trial variability in drift rate *s*_*v*_, the selected model was a mixture of a zero-bounded truncated normal and a gamma distribution (wAIC = 0.35), where the truncated normal had the highest mixture weight. Lastly, for *s*_*z*_*r*__, the selected model was a truncated normal distribution on [0, 1] (wAIC = 0.74).

## 4. Discussion

The increasing popularity of cognitive modeling has led to extensive applications of models like the Diffusion Decision Model (DDM) across a range of disciplines. These applications have the potential to provide substantial information about the plausible values of the parameters of cognitive models. We believe that for cognitive models where sufficient information are available in the literature, a systematic quantitative characterization of model parameters can be a very useful addition to existing modeling practices. Parameter reviews can benefit modeling practices in various ways, from facilitating parameter estimation to enabling more precise and realistic simulations to improve study design and calibrate future experiments (Gluth and Jarecki, 2019; Heck and Erdfelder, 2019; Pitt and Myung, 2019).

Here, we used the DDM as example case of how a systematic quantitative parameter review can be incorporated into modeling practices to provide informative prior distributions for the model parameters. Our empirical distributions of the parameter estimates were largely consistent with those of Matzke and Wagenmakers (2009), but because our sample was much larger we were better able to capture the tails of the parameter distributions. Although, for simplicity, here we suggested single-component distributions as priors, the full mixture distributions that we selected could also be used. Bayesian DDM software, such as the Dynamic Models of Choice software (DMC; Heathcote et al., 2019), can be easily adapted to use any form of univariate prior, including mixtures. In most cases the mixture served to inflate the distributions' tails while preserving a single mode. However, aggregation over heterogeneous studies naturally carries with it the possibility of creating multi-modal prior distributions, as illustrated by the results for *s*_*v*_ in Figure 3. If the data proved sufficiently uninformative that such multi-modality carried through to the posterior, caution should be exercised in reporting and interpreting measures of central tendency.

Inferring the parameters of complex cognitive models like the DDM from experimental data is challenging because their parameters are often highly correlated. The cumulative knowledge distilled into parameter estimates from past research can practically benefit both traditional optimization-based methods (e.g., maximum likelihood) and Bayesian estimation. In the former case, parameter reviews can provide informed guesses for optimization starting points as well as guidance for configuring bounded optimization methods. Even when powerful and robust optimization algorithms (e.g., particle swarm methods) are used, reasonable initial values and bounds can increase time efficiency and are often helpful for avoiding false convergence on sub-optimal solutions. In the latter—Bayesian case—parameter reviews can facilitate the use of informative prior distributions, which benefits both Bayesian model selection and parameter estimation.

Informative priors are essential for Bayesian model selection using Bayes factors (Jeffreys, 1961; Kass and Raftery, 1995). Unlike other model-selection methods like the Deviance Information Criteria (DIC; Spiegelhalter et al., 2014) and Widely Applicable Information Criterion (WAIC; Vehtari et al., 2017) that depend only on posterior samples, Bayes factors depend crucially on the prior distribution even when large amounts of data are available. This is because the marginal likelihood of the competing models is obtained by taking a weighted average of the probability of the data across all possible parameter settings with the weights given by the parameters' prior density. The workflow outlined here may therefore facilitate the more principled use of prior information in Bayesian model selection in the context of evidence-accumulation models (for recent developments, see Evans and Annis, 2019; Gronau et al., 2020).

In terms of Bayesian estimation, the extra constraint provided by informative priors can benefit some parameters more than others. In the DDM, for example, the across-trial variability parameters are notoriously difficult to estimate (Boehm et al., 2018; Dutilh et al., 2019). This has led to calls for these parameters to be fixed to zero (i.e., use the simple diffusion model; Stone, 1960) to improve the detection of effects on the remaining parameters (van Ravenzwaaij et al., 2017). Informative priors may provide an alternative solution that avoids the potential systematic distortion caused by ignoring the variability parameters (Ratcliff and McKoon, 2008) and enables the study of effects that cannot be accommodated by the simple diffusion model, such as differences between correct and error RTs (Damaso et al., submitted). Of course, in extreme cases, the central tendency of informative prior distributions may provide guidelines for fixing difficult-to-estimate model parameters to a constant (e.g., Matzke et al., 2020).

Information about the empirical distribution of parameter estimates, both in terms of the main body and the tails of the distributions, can especially benefit design optimization and parameter estimation in non-standard and difficult to access populations (e.g., Shankle et al., 2013; Matzke et al., 2017). For example, in clinical populations long experimental sessions are often impossible due to exhaustion or attention lapses. Expenses can also be constraining, such as with studies using costly fMRI methods. Therefore, data are often scarce, with a total number of trials as low as 100 reported in some DDM applications (e.g., O'Callaghan et al., 2017). In these cases, experimental designs can be optimized, and parameter estimation improved, with the aid of informative parameter distributions that put weight on plausible parts of the parameter space. Moreover, informative priors can also increase sampling efficiency and speed up the convergence of MCMC routines.

Ideally, informative prior distributions for cognitive models should be based on prior information extracted from experimental paradigms (or classes of paradigms) and participant populations relevant to the research question at hand, although care should be exercised in the latter case where group members fall along a continuum (e.g., age or the severity of a clinical diagnosis). In reality, constructing such highly specific priors might not always be feasible, either because of a paucity of relevant parameter values reported in the literature, or when new paradigms or populations are studied. However, we believe that using informative priors based on a range of broadly similar paradigms and heterogeneous populations is better than using vague priors, as long as appropriate caution is exercised in cases when the data are not sufficiently informative and hence the prior dominates inferences about the model parameters. Here, we presented informative prior distributions for parameter estimates aggregated across paradigms and populations and also provided paradigm-specific priors for the two most popular tasks in our database (e.g., lexical decision and random dot motion task) and priors for non-clinical populations.

The variance of the paradigm/population-specific priors showed a general decreasing tendency. The decrease in variance was relatively small for the priors based on non-clinical populations, and was restricted to the main DDM parameters. For the lexical decision task, the variance of all of the priors decreased relative to the overall priors. For the random dot motion task, with the exception of *v*, all variances decreased, albeit the decrease was negligible for *a*. To summarize, for the tasks and groups we examined here, paradigm and population heterogeneity appears to introduce additional variability in the parameter estimates, but the degree of additional variability strongly depends on the type of parameter. Our open repository provides the data and code to generate informed prior distributions for any selection of studies included in the database so that researchers can construct informative prior distributions relevant to their own research questions. Naturally, paradigm/population-specific priors are only sensible when a sufficiently large number of parameter estimates are available in the database to fit the theoretical (mixture) distributions, or when researchers can augment the repository with estimates from additional studies.

Despite their usefulness, systematic quantitative parameter reviews are not without their pitfalls. Using available cumulative knowledge from past literature always has to be viewed in light of the file drawer problem (Rosenthal, 1979). Many researchers have not published their non-significant results, therefore the literature is biased, and thus the parameter estimates retrieved from the literature might be biased toward specific model settings that converged or led to significant results. Furthermore, some cognitive models are too new and have not been widely applied to empirical data, so past literature might not provide researchers with a sufficiently reliable representation of the distribution of the parameter estimates. Therefore, cognitive modelers may not always be able to incorporate our proposed quantitative parameter review into their workflow, and should carefully weigh out the feasibility and benefits of such an endeavor.

### 4.1. Recommendations for Reporting Cognitive Modeling Results

Our literature review revealed a wide variety of reporting practices, both in terms of *what* researcher report and *how* they report their modeling results. The diversity of reporting practices is likely to reflect differences between disciplines and is in itself not problematic. However, we believe that the full potential of cumulative science can only be realized if authors provide sufficient information for others to interpret and reproduce their results. We endorse code and data sharing, and—following Lee et al. (2019)—we strongly urge researchers to provide sufficiently precise mathematical and statistical descriptions of their models, and to post-register exploratory model developments. In what follows, we reflect on the challenges we faced in performing the systematic parameter review, and formulate a set of general and DDM-specific suggestions that aim to increase computational reproducibility and the expected information gain from parameter reviews. Although our recommendations are certainly not exhaustive and do not apply to all model classes, we hope that they provide food for thought for cognitive modelers in general and RT modelers in particular.

#### 4.1.1. Model Parameterization and Scaling

The following recommendations are aimed at supporting well-informed choices about which model and which model parameters to include in a parameter review. Most parametric cognitive models can be parameterized in various ways. First, some cognitive models require fixing one (or more) parameters to make the model identifiable (Donkin et al., 2009b; van Maanen and Miletić, 2020). In the DDM, modelers typically fix the moment-to-moment variability of drift rate *s* to 0.1 or 1 for scaling purposes. Note, however, that the exact value of the scaling parameter is arbitrary, and—depending on the application—one may chose to estimate *s* from the data and use other parameters for scaling. We stress the importance of explicitly reporting which parameters are used for scaling purposes and the value of the scaling parameter(s) because the chosen setting influences the magnitude of the other parameter estimates. Another scaling issue relates to the measurement units of the data. For example, RTs are commonly measured in both seconds and milliseconds. Although the measurement scale influences the magnitude of the parameter estimates, none of the articles included in the present parameter review explicitly reported the measurement unit of their data. Further, articles did not consistently report all parameter estimates on the same RT scale (i.e., all parameter estimates reported in seconds, but *T*_*er*_ reported in milliseconds). Hence, we urge researchers to make an explicit statement on this matter and whenever possible stick to the same measurement unit throughout an article to avoid any ambiguity.

Second, in cognitive models one parameter is sometimes expressed as a function of one or more other parameters. The DDM, for instance, can be parameterized in terms of *absolute* starting point *z* or *relative* starting point $z_{r}=\frac{z}{a}$ (i.e., bias). The choice between *z* and *z*_*r*_ depends on the application but can also reflect default software settings. Although the two parametrizations are mathematically identical and have no consequences for the magnitude of the other parameters, it is clearly important to communicate which parameterization is used in a given application. Third, in many applications, researchers impose constraints on the model parameters across experimental manipulations, conditions, or groups. Such constraints sometimes reflect practical or computational considerations, but preferably they are based on a priori theoretical rationale (e.g., threshold parameters cannot vary based on stimulus properties that are unknown before a trial commences; Donkin et al., 2009a) or the results of model-selection procedures (e.g., Heathcote et al., 2015; Strickland et al., 2018). Regardless of the specific reasons for parameter constraints, we urge modelers to clearly communicate which parameters are hypothesized to reflect the effect(s) of interest, and so which are fixed and which are free to vary across the design. Moreover, we recommend researchers to report the competing models (including the parametrization) that were entertained to explain the data, and indicate the grounds on which a given model was chosen as best, such as AIC (Akaike, 1981), BIC (Schwarz, 1978), DIC (Spiegelhalter et al., 2002, 2014), WAIC (Watanabe, 2010), or Bayes factors (Kass and Raftery, 1995). We note that parameter reviews are also compatible with cases where there is uncertainty about which is the best model, through the use of Bayesian model averaging (Hoeting et al., 1999). In this approach, the parameter estimates used in the review are averaged across the models in which they occur, weighted by the posterior probability of the models.

#### 4.1.2. Model Estimation

In the face of the large number of computational tools available to implement cognitive models and the associated complex analysis pipelines, researchers have numerous choices on how to estimate model parameters. For instance, a variety of DDM software is available, such as fast-DM (Voss and Voss, 2007), HDDM (Wiecki et al., 2013), DMC (Heathcote et al., 2019), DMAT (Vandekerckhove and Tuerlinckx, 2008), using a variety of estimation methods, such as maximum likelihood, Kolmogorov-Smirnov, chi-squared minimization (Voss and Voss, 2007), quantile maximum probability (Heathcote and Brown, 2004), or Bayesian Markov chain Monte Carlo (MCMC; e.g., Turner et al., 2013) techniques. We encourage researchers to report the software they used, and whenever possible, share their commented code to enable computational reproducibility (McDougal et al., 2016; Cohen-Boulakia et al., 2017). Knowledge about the estimation software can also provide valuable information about the parametrization and scaling issues described above.

#### 4.1.3. Parameter Estimates and Uncertainty

We recommend researchers to report all parameter estimates from their chosen model and not only the ones that are related to the experimental manipulation or the psychological effect of interest. In the DDM in particular, this would mean reporting the across-trial variability parameters, and not only the main parameters (i.e., drift rate, boundary separation, starting point, and non-decision time), even if only a subset of parameters is the focus of the study. Ideally, in the process of aggregation used to create prior distributions, estimates should be weighted by their relative uncertainty. The weighing should reflect the uncertainty of the individual estimates resulting from fitting the model to finite data and—if average parameters are used, as was the case here—also sampling error reflecting the sample size used in each study. Although we had access to the sample sizes, most studies reported parameter estimates averaged across participants without accounting for the uncertainty of the individual estimates. Moreover, the few studies that reported individual estimates provided only point estimates and failed to include measures of uncertainty. As a proxy to participant-level measures of uncertainty one may use the number of trials that provide information for the estimation of the various model parameters. However, this approach requires a level of detail about the experimental design and the corresponding model specification (including the number of excluded trials per participant) that was essentially never available in the surveyed studies.

Given these problems with reporting, we have decided to give equal weights to all (averaged) parameter estimates regardless of the sample size. The reason for this decision was that studies with large sample sizes typically used a small number of trials and likely resulted in relatively imprecise individual estimates, whereas studies with small sample sizes typically used a large number of trials and likely resulted in relatively precise individual estimates. We reasoned that as a result of this trade-off, the equal weighting may not be necessarily unreasonable. To remedy this problem in future parameter reviews, we urge researchers to either report properly weighted group average estimates or report individual estimates along with measures of uncertainty, let these be (analytic or bootstrapped) frequentist standard errors and confidence intervals (e.g., Visser and Poessé, 2017), or Bayesian credible intervals and full posterior distributions (Jeffreys, 1961; Lindley, 1965; Eberly and Casella, 2003).

#### 4.1.4. Individual Parameters and Correlations

Ideally, researchers should report parameter estimates for each individual participant. In the vast majority of the studies examined here, only parameters averaged over participants were available. This means that we were unable to evaluate correlations among parameter estimates reflecting individual differences. Such correlations are likely quite marked. For example, in the DDM a participant with a higher drift rate, which promotes accuracy, is more likely to be able to afford to set a lower boundary and still maintain good performance, so a negative correlation between rates and boundaries might be expected. Access to individual parameters would allow estimation of these correlations, and thus enable priors to reflect this potentially important information. As we discuss below, the failure to report individual estimates brings with it important limitations on what can be achieved with the results of systematic parameter reviews.

### 4.2. Limitations and Future Directions

The approach to parameter reviews taken here—obtaining values from texts, tables, and graphs from published papers and performing an aggregation across studies—has the advantage of sampling estimates that are representative of a wide variety of laboratories, paradigms, and estimation methods. Indeed, for the priors presented in Figure 3 we included a few studies with much longer RTs than are typically fit with the DDM (e.g., Lerche and Voss, 2019). The larger parameter values from these studies had the effect of broadening the tails of the fitted distributions so they represent the full variety of estimates reported in the literature.

However, this approach has a number of limitations beyond those related to the vagaries of incomplete reporting practices just discussed. The first limitation is related to the aggregation of parameter estimates over different designs. The most straightforward example concerns including parameters from studies with long RTs. The solution is equally straightforward: only including studies with RTs that fall in the range of interest specific to a particular application. A related but more subtle issue occurs in our DDM application where the meaning of the magnitude of the response bias (*z*_*r*_) parameter is design specific, and so it is difficult to form useful aggregates over different paradigms. To take a concrete example, a bias toward “word” responses over “non-word” responses in a lexical-decision paradigm cannot be made commensurate with a bias favoring “left” over “right” responses in a random dot motion paradigm. Our approach—forming an aggregate with maximum uncertainty by assuming either direction is equally likely (i.e., mirroring the values)—removes any information about the average direction while at least providing some information about variability in bias. Although this approach likely overestimates the variability of the bias estimates, we believe that overestimation is preferable to underestimation which might result in an overly influential prior distribution. Again, this problem can again be avoided by constructing priors based on a more specific (in this case task-specific) aggregation. Our online data repository reports raw starting point and bias estimates, which combined with the design descriptions from the original papers could be used to perform such an aggregation. The priors for the lexical decision task reported in the Supplementary Materials provide an example that did not require us to mirror the bias estimates. We note, however, that we had to exclude a paper where it was unclear which response was mapped to which DDM boundary, so we would add a reporting guideline that this choice be spelled out. We also note that similar problems with aggregation over different designs are likely to occur for other parameter types and also beyond the DDM, for instance in evidence-accumulation models such as the Linear Ballistic Accumulator (Brown and Heathcote, 2008). For instance, if one decomposes drift rates in the DDM into the average over stimuli and “stimulus bias” (i.e., the difference in rates between the two stimulus classes; White and Poldrack, 2014), then the same issue applies, but now with respect stimuli rather than responses.

The second limitation—which is related to incomplete reporting, but is harder to address within a traditional journal format—concerns obtaining a full multivariate characterization of the prior distribution of parameters that takes into account correlations among parameters as well as their average values and variability. Because most estimates reported in the literature are averages over participants, we were restricted to providing separate univariate characterizations of prior distributions for each parameter. To the degree that the implicit independence assumption of this approach is violated^5^ problems can arise. Continuing the example of negatively correlated rates and boundaries, although a higher value of both separately may be quite probable, both occurring together may be much less likely that the product of their individual probabilities that would be implied by independence.

Problems related to this limitation arise, for example, if in planning a new experiment one were to produce synthetic data by drawing parameter combinations independently from the univariate priors in Figure 3, potentially producing simulated participants with parameter values that are unlikely in a real experiment. With Bayesian methods, ignoring the correlations among parameters can compromise the efficiency of MCMC samplers and complicate the interpretation of Bayes factors because the resulting uni-variate priors will assign mass to implausible regions of the parameter space. Although standard Bayesian MCMC samplers used for evidence-accumulation models have not taken account of these population correlations, a new generation of samplers is appearing that does (Gunawan et al., 2020). This development underscores the need for future systematic parameter reviews to move in the direction of multivariate characterizations. This may be achieved by revisiting the original data sets, which due to open science practices are becoming increasingly available, refitting the DDM, and then using the resulting individual parameter estimates to form multivariate priors. This future direction will be time consuming and computationally challenging, and will no doubt bring with it new methodological problems that we have not addressed here. Nevertheless, we believe that the long-term gains for cognitive modeling will make this enterprise worthwhile.

## Data Availability Statement

The datasets presented in this study can be found in online repositories. The names of the repository/repositories and accession number(s) can be found below: https://osf.io/9ycu5/and GitHub: https://github.com/nhtran93/DDM_priors.

## Author Contributions

N-HT designed the study, collected the data, performed the analyses, and wrote the manuscript. N-HT, LvM, and DM designed the study and the analyses. DM and AH provided critical feedback and helped shape the manuscript. All authors contributed to the article and approved the submitted version.

## Conflict of Interest

The authors declare that the research was conducted in the absence of any commercial or financial relationships that could be construed as a potential conflict of interest.

## Footnotes

^1^Based on personal communication with Roger Ratcliff.

^2^Based on personal communication with Roger Ratcliff.

^3^The bias *z*_*r*_ parameters estimated using the HDDM software (Wiecki et al., 2013) are coded as 1 − *z*_*r*_ in our parameter review. Note that this has no influence on the resulting prior distribution as we used both *z*_*r*_ and 1 − *z*_*r*_ to create the prior.

^4^The location and scale parameters of the truncated normal distribution refer to μ and σ and not to its expected value and variance.

^5^To be clear, we are not talking about correlations among parameters within a participant, which are a consequence of the mathematical form of the model's likelihood and the particular parameterization adopted for the design. Rather, we are addressing correlations at the population level, i.e., across participants. Although the two types of correlations can be related, they are not the same and in our experience can sometimes differ very markedly.
