# Supplementary material for: Systematic Parameter Reviews in Cognitive Modeling: Towards a Robust and Cumulative Characterization of Psychological Processes in the Diffusion Decision Model
Source: Front Psychol. 2021 Jan 21;11:608287. doi: 10.3389/fpsyg.2020.608287 (PMC7874054; doi:10.3389/fpsyg.2020.608287)
Supplement: Supplementary file 1 [file Data_Sheet_1.pdf]

Supplementary Materials for "Systematic Parameter Reviews in Cognitive Modeling: Towards a Robust and Cumulative Characterization of Psychological Processes in the Diffusion Decision Model"

1 SEARCH QUERIES FOR LITERATURE SEARCH

The following search strings were used:

- PsycInfo: ((drift diffusion model* or diffusion decision model or ratcliff diffusion or diffusion model) and (response time* or reaction time*)).mp. and Reaction time.sh. limit 1 to yr="1977 - 2017"
- Pubmed: ((((diffusion[Text Word] OR drift*[Text Word] OR *DDM[Text Word]) AND model*[Text Word]) AND ("1978"[Date - Publication] : "2017"[Date - Publication])) AND "Reaction time"[MeSH Terms])
- Web of Science: (TS=("drift diffusion model") OR TS=("diffusion decision model") OR TS=("ratcliff diffusion") OR TS=("diffusion model")) AND (TS=(response time* OR reaction time*)) AND (SU=(Life Sciences Biomedicine) OR SU=(Social Sciences)) Indexes=SCI-EXPANDED, SSCI, A&HCI, ESCI Timespan=1977-2017
- Scopus: (ALL ("response* time*") OR ALL ("reaction* time*")) AND (TITLE-ABSKEY ("diffusion model") OR TITLE-ABS-KEY ("drift diffusion model") OR TITLE-ABS-KEY ("*DDM") OR TITLE-ABS-KEY ("diffusion decision model") OR TITLE-ABS-KEY ("Ratcliff diffusion")) AND PUBYEAR > 1977 AND SUBJAREA (psyc OR neur OR medi OR soci OR deci OR econ OR mult)

2 PARAMETER TRANSFORMATIONS

The reported parameter estimates were re-scaled to an RT scale in seconds and moment-to-moment variability in drift rate s of 1 as follows:

$$\begin{aligned}
v &= v \times \frac{\sqrt{\text{scaling}}}{\text{scaling}} \\
a &= a \times \sqrt{\text{scaling}} \\
z &= z \times \sqrt{\text{scaling}} \\
T_{er} &= T_{er} \times \text{RT scaling} \\
s_v &= s_v \times \frac{\sqrt{\text{scaling}}}{\text{scaling}} \\
s_z &= s_z \times \sqrt{\text{scaling}} \\
s_{T_{er}} &= s_{T_{er}} \times \text{RT scale},
\end{aligned}$$

where scaling $=$ RT scaling $\times$ s scaling. For instance, suppose we want to re-scale parameter estimates from milliseconds to seconds and from $s = 0.1$ to $s = 1$. The scaling factor would be computed as scaling $= \frac{1}{1000} \times 10$. Bias $z_r = \frac{z}{a}$ and $s_{z_r} = \frac{s_z}{a}$ require no scaling. The value of non-decision time T_{er} and $s_{T_{er}}$ are not affected by the value of s, therefore, only the RT scaling is necessary.

3 PARADIGM–SPECIFIC INFORMATIVE PRIOR DISTRIBUTIONS

We provide two examples of paradigm–specific priors. We chose the two most common tasks in our database: the lexical decision task (33 articles) and the random dot motion task (18 articles). We generated informative prior distributions according to the methodology described in the main text, with the following modifications.

1. With one exception, all lexical decision studies mapped "word" responses onto the upper decision boundary, so z_r reflects a bias towards "word" responses over "non-word" responses. In the remaining case, the article did not provide sufficient information to determine the mapping between response options and boundaries. After excluding this single study, we collapsed all z_r estimates in a distribution, but —unlike in the main analyses where we treated the assignment of responses to boundaries as arbitrary — we did not mirror the distribution of z_r.
2. As our database contained only 18 random dot motion studies, the number of data points for z_r and the across-trial variability parameters was very low with only 10 or even fewer estimates. Therefore, we only constructed prior distributions for v, a, and T_{er}.

The resulting prior distributions for the lexical decision task and the random dot motion task are shown in Table S1 and Figure S1, and Table S2 and Figure S2, respectively.

Table S1. Informative Priors Distributions for the Lexical Decision Task

DDM Parameter	N	Distribution	Weight	Location/Shape	Scale	df	T-LB	T-UB	E-LB	E-UB
v	382	truncated normal* & truncated normal	0.55	2.33	0.58		0	+ Inf	0.06	8.73
a	130	lognormal* & truncated normal	0.76	0.32	0.32		0	+ Inf	0.74	3.93
z_r	38	truncated t	–	0.52	0.01	0.67	0	1	0.31	0.77
T_{er}	152	truncated normal* & lognormal	0.53	0.44	0.03		0	+ Inf	0.35	0.90
s_v	83	truncated normal* & gamma	0.89	1.16	0.58		0	+ Inf	0.01	2.68
s_{z_r}	69	truncated normal	–	0.34	0.19		0	1	0.01	0.70
$s_{T_{er}}$	83	gamma* & truncated normal	0.94	17.37	0.01		0	+ Inf	0	0.83

Note. N: The number of unique estimates; Weight: The mixture weight of the dominant distribution component; df: degrees of freedom; T-LB: theoretical lower bound of the prior distribution; T-UB: theoretical upper bound of the prior distribution.; E-LB: lower bound of the empirical parameter estimates; E-UB: upper bound of the empirical parameter estimates; *: dominant distribution component.

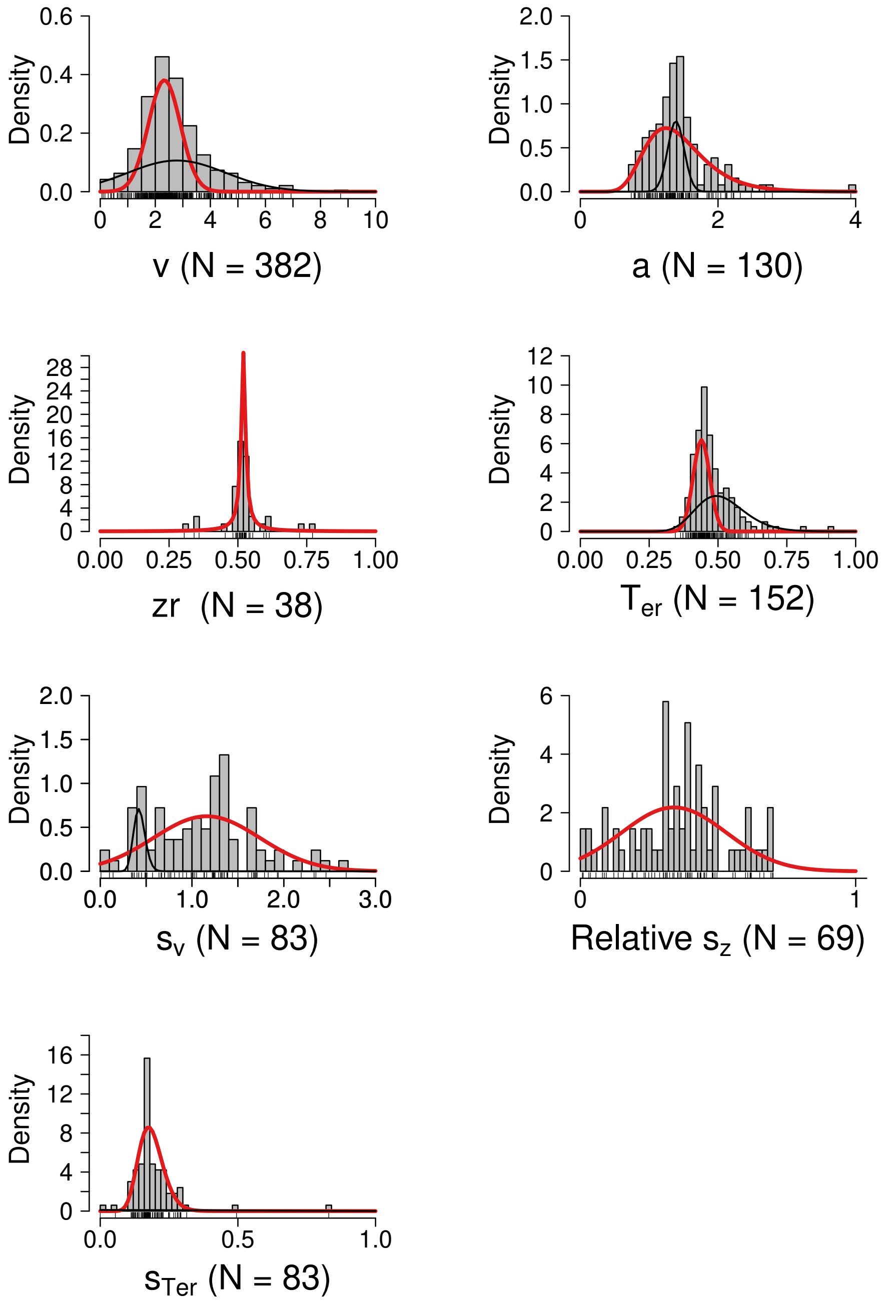

Figure S1. *Prior Distributions for the Lexical Decision Task*. The red lines show the best fitting theoretical distributions or the dominant theoretical distribution components with the highest mixture weight (i.e., the proposed informative prior distributions). The black lines show the non-dominant distribution components. N: number of unique estimates.

Table S2. Informative Priors Distributions for the Random Dot Motion Task

DDM Parameter	N	Distribution	Weight	Location/Shape	Scale	T-LB	T-UB	E-LB	E-UB
v	144	truncated normal	–	-207.1	25.88	0	+ Inf	0.01	17.65
a	95	gamma* & gamma	0.89	12.19	0.10	0	+ Inf	0.50	5.70
T_{er}	86	Weibull	–	5.12	0.44	0	+ Inf	0.00	0.56

Note. N: The number of unique estimates; Weight: The mixture weight of the dominant distribution component; df: degrees of freedom; T-LB: theoretical lower bound of the prior distribution; T-UB: theoretical upper bound of the prior distribution.; E-LB: lower bound of the empirical parameter estimates; E-UB: upper bound of the empirical parameter estimates; *: dominant distribution component.

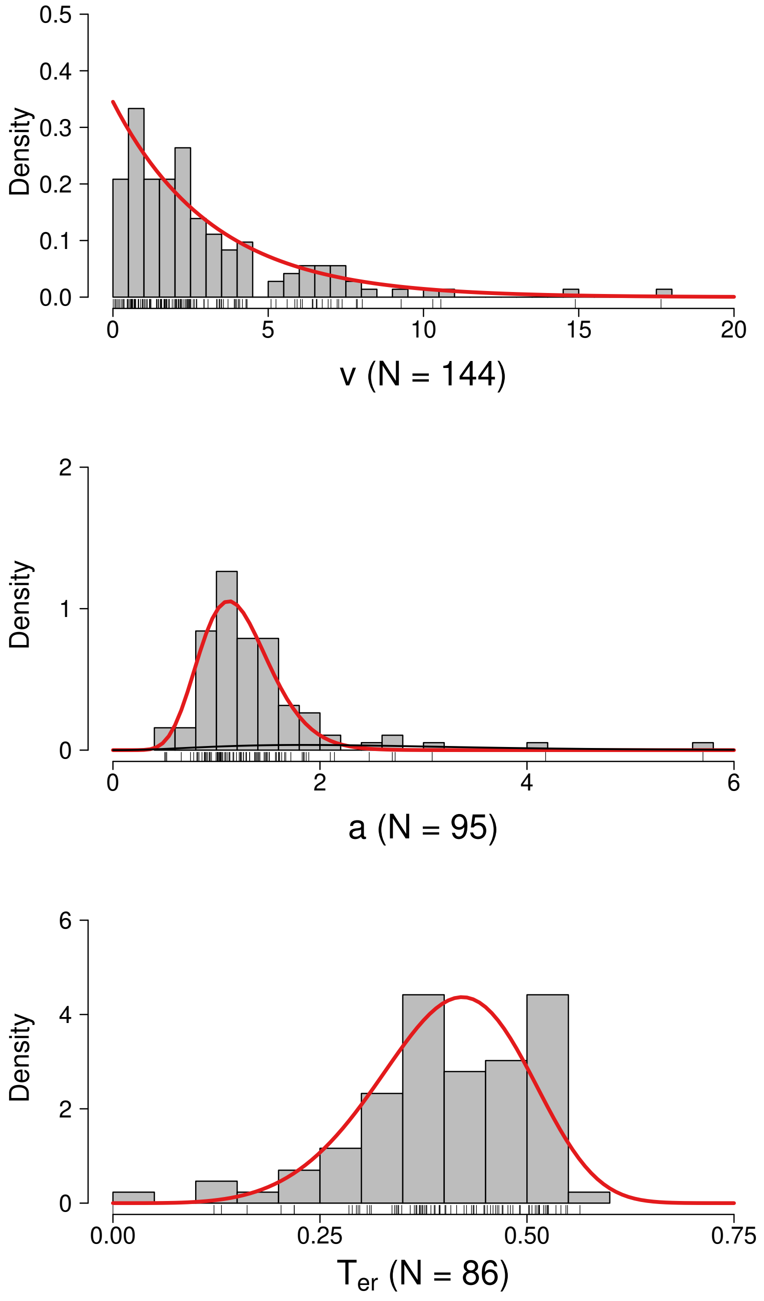

Figure S2. *Prior Distributions for the Random Dot Motion Task.* The red lines show the best fitting theoretical distributions or the dominant theoretical distribution components with the highest mixture weight (i.e., the proposed informative prior distributions). The black lines show the non-dominant distribution components. N: number of unique estimates.

4 INFORMATIVE PRIOR DISTRIBUTIONS FOR NON-CLINICAL POPULATIONS

Here we provide informative prior distributions for applications with healthy populations, after excluding the 29 clinical studies from the database. We generated informative prior distributions according to the methodology described in the main text. The resulting prior distributions are shown in Table S3 and Figure S3.

Table S3. Informative Priors Distributions for non-clinical Populations

DDM Parameter	N	Distribution	Weight	Location/Shape	Scale	df	T-LB	T-UB	E-LB	E-UB
v	1579	gamma* & gamma	0.65	1.39	1.72		0	+ Inf	0.01	13.79
a	731	truncated normal* & lognormal	0.73	1.24	0.40		0	+ Inf	0.11	6.80
Mirrored z_r	164	truncated t	–	0.5	0.04	2.65	0	1	0.04	0.96
T_{er}	718	gamma* & gamma	0.86	22.17	0.02		0	+ Inf	0.00	3.69
s_v	282	truncated normal* & gamma	0.79	1.32	0.72		0	+ Inf	0.00	3.45
s_{z_r}	248	truncated normal	–	0.33	0.22		0	1	0.01	0.85
$s_{T_{er}}$	318	truncated normal* & lognormal	0.80	0.17	.05		0	+ Inf	0	4.75

Note. N: The number of unique estimates; Weight: The mixture weight of the dominant distribution component; df: degrees of freedom; T-LB: theoretical lower bound of the prior distribution; T-UB: theoretical upper bound of the prior distribution.; E-LB: lower bound of the empirical parameter estimates; E-UB: upper bound of the empirical parameter estimates; *: dominant distribution component.

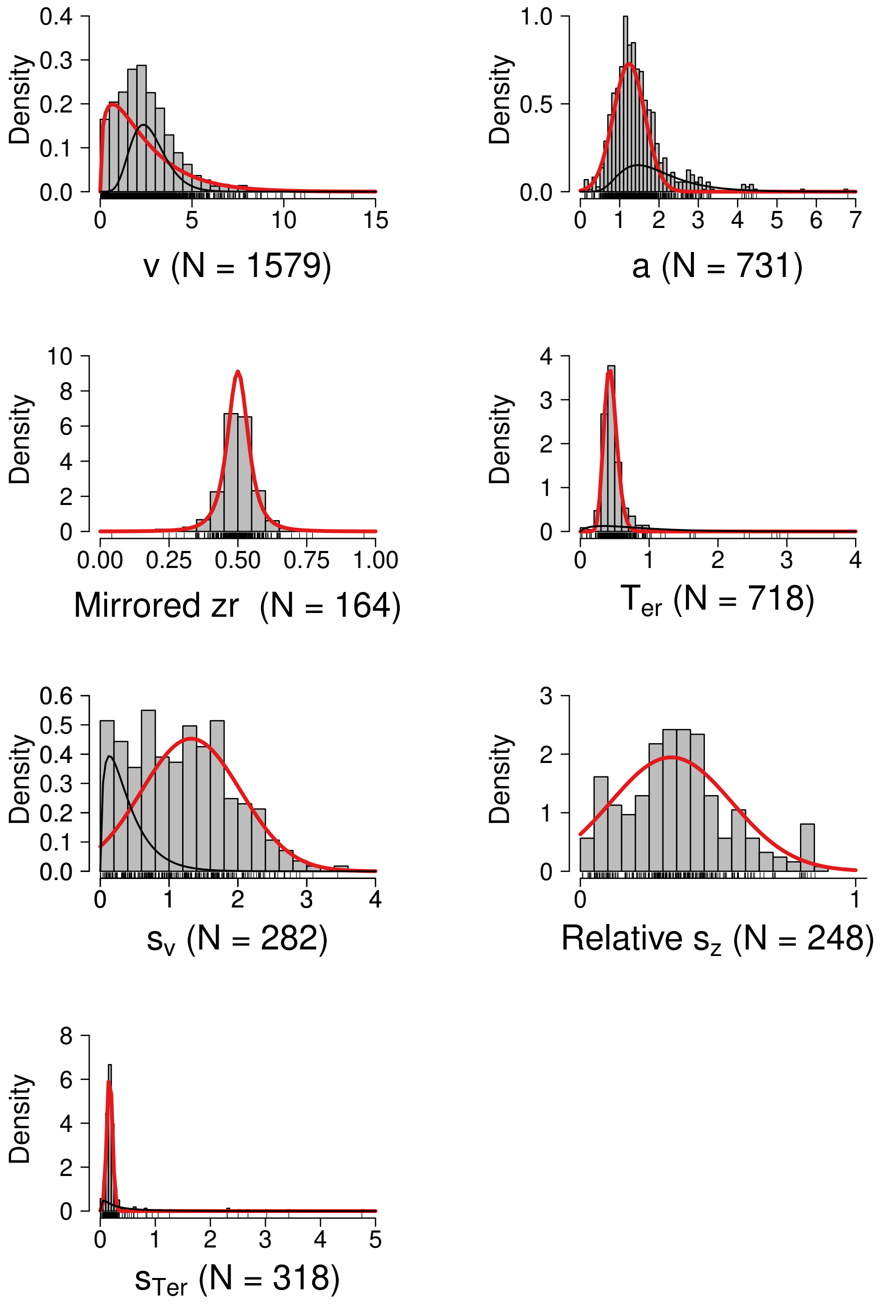

Figure S3. *Prior Distributions for Non-Clinical Populations*. The red lines show the best fitting theoretical distributions or the dominant theoretical distribution components with the highest mixture weight (i.e., the proposed informative prior distributions). The black lines show the non-dominant distribution components. N: number of unique estimates.
